# Musical memories in newborns: A resting‐state functional connectivity study

**DOI:** 10.1002/hbm.25677

**Published:** 2021-11-05

**Authors:** Serafeim Loukas, Lara Lordier, Djalel‐Eddine Meskaldji, Manuela Filippa, Joana Sa de Almeida, Dimitri Van De Ville, Petra S. Hüppi

**Affiliations:** ^1^ Division of Development and Growth, Department of Pediatrics University of Geneva Geneva Switzerland; ^2^ Institute of Bioengineering, Ecole Polytechnique Fédérale de Lausanne (EPFL) Lausanne Switzerland; ^3^ Department of Radiology and Medical Informatics University of Geneva Geneva Switzerland; ^4^ Institute of Mathematics, Ecole Polytechnique Fédérale de Lausanne (EPFL) Lausanne Switzerland

**Keywords:** brain networks, connectomics, engrams, functional connectivity, musical memories, prematurity, resting‐state fMRI

## Abstract

Music is known to induce emotions and activate associated memories, including musical memories. In adults, it is well known that music activates both working memory and limbic networks. We have recently discovered that as early as during the newborn period, familiar music is processed differently from unfamiliar music. The present study evaluates music listening effects at the brain level in newborns, by exploring the impact of familiar or first‐time music listening on the subsequent resting‐state functional connectivity in the brain. Using a connectome‐based framework, we describe resting‐state functional connectivity (RS‐FC) modulation after music listening in three groups of newborn infants, in preterm infants exposed to music during their neonatal‐intensive‐care‐unit (NICU) stay, in control preterm, and full‐term infants. We observed modulation of the RS‐FC between brain regions known to be implicated in music and emotions processing, immediately following music listening in all newborn infants. In the music exposed group, we found increased RS‐FC between brain regions known to be implicated in familiar and emotionally arousing music and multisensory processing, and therefore implying memory retrieval and associative memory. We demonstrate a positive correlation between the occurrence of the prior music exposure and increased RS‐FC in brain regions implicated in multisensory and emotional processing, indicating strong engagement of musical memories; and a negative correlation with the Default Mode Network, indicating disengagement due to the aforementioned cognitive processing. Our results describe the modulatory effect of music listening on brain RS‐FC that can be linked to brain correlates of musical memory engrams in preterm infants.

## Graphical abstract

Using a connectome‐based framework, we describe resting‐state functional connectivity (RS‐FC) modulation after music listening in three groups of newborn infants, in preterm infants exposed to music during their neonatal‐intensive‐care‐unit (NICU) stay, in control preterm, and full‐term infants. Our results describe the modulatory effect of music listening on brain RS‐FC that can be linked to brain correlates of musical memory engrams in preterm infants.

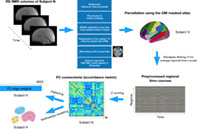

## 1 INTRODUCTION

Understanding the preterm infants' brain maturity and its functional integrity is pivotal for developing new individually oriented care interventions, such as music for preterm infants in the neonatal intensive care unit (NICU). Resting‐state functional magnetic resonance imaging allows the assessment of functional connectivity (RS‐FC) between resting‐state networks (RSNs), and is an ideal way to study the functional networks of the preterm infants' brain. In fact, functional connectivity at rest has been shown to provide information about basic brain processing, brain maturity and integrity (Bäuml et al., 2015; Dosenbach et al., 2010; Pruett et al., 2015). Consistent RSNs are found across healthy adults, and most of these networks are also observed in children, infants, and neonates (Doria et al., 2010; Lordier et al., 2019; Thornburgh et al., 2017). These RSNs have been linked to brain functions, such as sensorimotor, visual, auditory, as well as higher‐order cognitive processes (Doucet et al., 2011; Reineberg, Andrews‐Hanna, Depue, Friedman, & Banich, 2015; Van Den Heuvel & Pol, 2010).

Even if RSNs are consistently present in healthy adults, a variation of the functional connectivity within and across them has been previously observed. Cognitive, motor, or music expertise has been linked to an increased RS‐FC between regions involved in the processing of the aforementioned expertise (for a review see: Cantou, Platel, Desgranges, & Groussard, 2018). A number of previous studies have compared RS‐FC in musicians and nonmusicians and found RS‐FC connectivity between limbic, sensorimotor, and auditory regions to be correlated with the number of years of practice (Palomar‐García, Zatorre, Ventura‐Campos, Bueichekú, & Ávila, 2017; Zamorano, Cifre, Montoya, Riquelme, & Kleber, 2017). Recently, we have shown that even a session of receptive music listening in NICU can provoke a functional network reorganization at term‐equivalent age (TEA) (Lordier et al., 2019). Higher RS‐FC was found in the music intervention group both in sensory functional networks and in networks implicated in higher‐level functions, namely between auditory, sensorimotor, thalamus, salience network, superior frontal, and precuneus networks. Hence, past musical experience has effects on brain RS‐FC development that can be observed at 40 weeks of gestational age in pre‐term infants.

In adults, RS‐FC is also influenced by a particular state of mind, mood, and preceding stimulus‐induced brain activity. It has been shown that hypnosis (Demertzi et al., 2011), acupuncture (Dhond, Yeh, Park, Kettner, & Napadow, 2008), sad mood induction (Bernat & Stępień, 2010; Harrison et al., 2008), stress (Van Marle, Hermans, Qin, & Fernández, 2010), anxiety (Simpson, Drevets, Snyder, Gusnard, & Raichle, 2001), emotion induction (Eryilmaz, Van De Ville, Schwartz, & Vuilleumier, 2011), and attention tasks (Elton & Gao, 2015) induce a variation on the subsequent RS‐FC in adults. Furthermore, modification of RS‐FC has also been observed after music listening in adults. In addition, pleasant and relaxing music was used to reduce pain perception in fibromyalgia patients (Garza‐Villarreal et al., 2015). To this end, patients were asked to listen to their preferred relaxing music. When comparing RS‐FC before and after the musical stimuli, a modulation of RS‐FC between brain regions related to music, pain, and analgesia processing was observed. The RS‐FC of the angular gyrus was especially modified after music listening, a region which is connected to parietal, temporal, and frontal regions, representing networks implicated in multisensory information integration (Seghier, 2013) and in memory, allowing the recognition of the music as familiar (Platel, Baron, Desgranges, Bernard, & Eustache, 2003). Listening to familiar, pleasant and soothing music may thus impact brain states and subsequent RS‐FC. Furthermore, in the present study, preterm infants had listened to the same musical composition during their entire stay in the NICU, and this music was presented prior to waking‐up or falling asleep and in moments of alertness to interact with the environment, which are considered to be meaningful salient events for these newborns. Indeed, when preterm infants listen to this same music during an fMRI experiment, brain regions implicated in emotionally arousing and familiar music processing, are activated (Lordier et al., 2018, 2018).

In this recent task‐based fMRI study, we observed brain functional connectivity modulation during music listening in newborns. We used Psychophysiological Interaction Analysis (PPI), which estimates context‐specific changes in coupling between a seed region and the rest of the brain, to argue that music listening during NICU stay improves music processing in preterm newborns (Lordier et al., 2018, 2018). When preterm infants listened to the known music (heard previously in the neonatology) compared to the same music played 40% faster (changing the tempo), the functional connectivity of the auditory cortex with putamen, caudate and thalamus was increased. We interpreted this increased connectivity both in terms of tempo processing expertise and of familiar music processing since these brain regions have been shown in adults to be activated more for familiar than for unfamiliar music (Freitas et al., 2018; Pereira et al., 2011) and for beat prediction than beat detection (Grahn & Rowe, 2013). We concluded that, due to the early music intervention, preterm newborns acquired expertise in music feature processing, and the music they heard during their hospitalization had become familiar to them (Lordier et al., 2018, 2018). Furthermore, familiarity is considered as a semantic memory process, that is, a long‐term storage of music (Groussard et al., 2010), and an implicit memory phenomenon dependent on the integrity of pitch and rhythm perception (Peretz, Gaudreau, & Bonnel, 1998). Also, adult musicians, compared to nonmusicians, have larger auditory working memory (Parbery‐Clark, Skoe, Lam, & Kraus, 2009). The familiarity effect that we observed in this previous experience may therefore be directly linked to an auditory memory effect.

Finally, learning tasks induce changes in subsequent resting‐state functional connectivity, which have been shown to be correlated with improvements in later task performance (Gregory et al., 2014). It is also worth noting that subsequent RS‐FC may not be increased only between brain regions activated during encoding, but also between these brain regions and regions implicated in later retrieval. Increased functional connectivity after a memory task has been observed between the hippocampus and cortical regions involved both in encoding and retrieval, and this RS‐FC predicted later memory performance (Tambini, Ketz, & Davachi, 2010). Additionally, after a memory task, Risius and colleagues (Risius et al., 2019) showed increased subsequent RS‐FC between brain regions activated during the task (including inferior occipital and fusiform gyri) and the middle temporal gyri, which was found to mediate later retrieval.

The question now being addressed is how music listening modifies the distributed brain networks in preterm and full‐term newborns, measured by the subsequent RS‐FC. We additionally hypothesize that preterm infants who were already exposed to music listening during their NICU stay would increase their RS‐FC between brain regions implicated in the formation and retrieval of musical memories. To this end, we used connectomic and multivariate techniques on the MRI based functional connectomes (Fornito, Zalesky, & Breakspear, 2015; Sporns, Tononi, & Kötter, 2005).

## 2 MATERIALS AND METHODS

### 2.1 Population

The present study was approved by the Research and Ethics Committees of the University Hospital of Geneva and informed written consent was obtained from the parents before participating in the study. Thirty‐nine preterm and 24 full‐term infants (FT) were recruited at Geneva University Hospital neonatal units. The preterm newborns were randomly assigned to either music intervention (PM: *n* = 20) or standard of care group (PC: *n* = 19). All infants underwent magnetic resonance imaging at 40 weeks gestational age (GA).

Inclusion criteria for full‐term newborns were birth after a GA of 37 weeks, an appropriate height, weight, and head circumference (above the 10th and below the 90th percentiles). Exclusion criteria for all babies were major brain lesions, such as high‐grade intraventricular hemorrhage or leukomalacia (1 PC and 2 FT were excluded). Four preterm and three full‐term infants dropped out of the study before completion of the MRI (3 PM, 1 PC, and 3 FT). Technical problems during the MRI prevented us from acquiring the necessary data for 1 preterm infant (1 PC). Furthermore, due to high levels of motion, RS‐fMRI data from six infants were not used in subsequent analyses (2 PM, 1 PC, and 3 FT)—see Figure S1.

The final analysis, after removal of motion corrupted MRI volumes, was performed on 15 premature babies in the PM group (eight females, mean (SD) GA at birth: 29.16 (2.14) weeks) scanned at TEA (mean (SD) GA at scan: 40.21 (0.55) weeks), 15 premature newborns in the PC group (eight females, mean (SD) GA at birth: 28.95 (1.83) weeks) scanned at TEA (mean (SD) GA at scan: 40.50 (0.77) weeks), and 16 FT (nine females, mean (SD) GA: 39.50 (1.08) weeks) scanned in the first 4 days of life (mean (SD) GA at scan: 39.78 (1.05) weeks). No statistically significant differences (using ANOVA for numerical and chi‐squared test for categorical variables) in gender, GA at birth, birth weight, birth height, head circumference at birth, neonatal asphyxia, chorioamnionitis, sepsis (positive blood culture), bronco‐pulmonary dysplasia (Jobe & Bancalari, 2001), intraventricular hemorrhages grade 1 and 2 (Papile, Munsick‐Bruno, & Schaefer, 1983), and mean number of music/no‐music intervention sessions were observed between the two preterm groups. Furthermore, no differences in gender, GA at scan and parental socioeconomic status (SES) were observed across the three groups. The population characteristics and the results of the aforementioned statistical tests are presented in Tables S1 and S5 in the “Statistical tests for clinical variables”.

### 2.2 MRI acquisition

Before the MRI, all infants received breast or formula feeding, were swaddled in a blanket, and set up in a vacuum pillow for immobilization. No sedation was used and the infants were scanned while resting quietly in the scanner. To protect infants from the noise of the scanner and to deliver the music, MR‐compatible headphones were used (MR confon, Magdeburg, Germany).

Two RS‐fMRI runs (run 1, run 2) of 8‐min duration were acquired resulting in 300 volumes using an EPI sequence with TR = 1,600 ms, TE = 30 ms, slice thickness = 3 mm, flip angle = 90°, matrix = 64 × 52, FOV = 160 × 130 mm, 30 slices and voxel size = 2.5 × 2.5 × 3 mm^3^ on a Siemens 3‐T scanner. Between these two RS‐fMRI runs, the music used for the musical intervention during the hospitalization was presented to all infants (three groups) in a task‐based fMRI run of 8 min. The musical stimulus was thus, familiar only to the Preterm‐Music group (PM). The two resting‐state sequences were acquired immediately before and after music listening and infants stayed in the scanner during the entire protocol. Also, T2‐weighted structural images were acquired for anatomical reference (113 coronal slices, TR = 4990 ms, TE = 151 ms, slice thickness = 1.2 mm, flip angle = 150°, matrix = 512 × 328, FOV = 128 × 200 mm and voxel size = 0.39 × 0.39 × 1.2 mm^3^). The acquisition protocol was identical for all three groups.

### 2.3 Music

The music was especially created by Andreas Vollenweider composed of a soothing background, bells, harp, and punji (charming snake flute) tones (http://vollenweider.com). This music was chosen based on behavioral responses of preterm newborns to the instruments (observed by a nurse specialized in developmental care) and its duration was 8 min. Extracts of this music were played to all groups between the two RS‐fMRI acquisitions.

The PM group underwent musical intervention during the hospitalization using headphones, five times per week (mean number: 25 ± 8.92, range: 7–35), from 33 weeks GA until the MRI scan (see Table S2 for more details). The control group had the same handling as the intervention group (namely putting headphones) but the headphones were made open to environmental sounds, as such representing an active control group. Music and no‐music sessions were administrated once a day, 5 days per week. For more details see Lordier et al., 2019.

### 2.4 Data preprocessing

**FIGURE 1:**
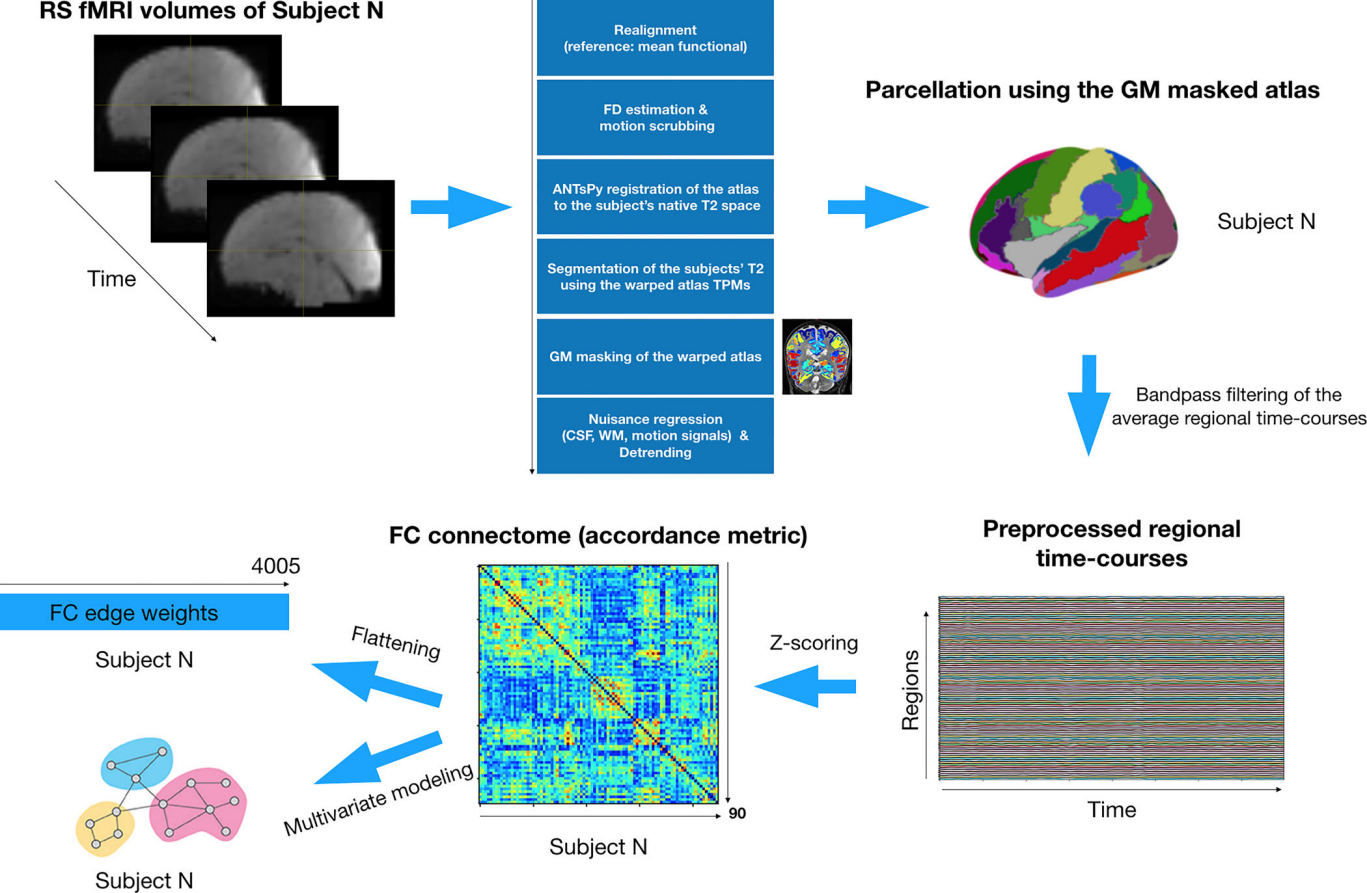
Flowchart showing the preprocessing and network construction pipeline. This procedure was repeated for each subject and each run

For each subject, the functional data were first realigned to the mean functional volume and then co‐registered to the structural image using Statistical Parametric Mapping software SPM12 [Wellcome Department of Imaging Neuroscience, (www.fil.ion.ucl.ac.uk/spm/software/spm12/)]. All volumes with a frame‐wise displacement (Power et al., 2014) >0.5 mm or with a rate of BOLD signal changes across the entire brain (spatial standard deviation of successive difference frames, DVARS) >3% were removed, along with the previous and the two successive images. The remaining images were included for further analysis. A minimum of 50% of volumes must have remained for inclusion. Significant differences in the number of excluded images were not observed between the three groups (average number of images removed ± SD; first MRI exam: FT: 29.6 ± 32.80 images, PM: 30 ± 32.68, PC: 27.73 ± 32.58; second MRI exam: FT: 48.67 ± 40.82 images, PM: 40.6 ± 32.54, PC: 31.07 ± 41.57). Six babies were excluded from subsequent analyses due to high levels of motion in data from run 1 and/or run 2. The preprocessing steps are summarized in the flowchart presented in Figure 1. Finally, the three groups were balanced in terms of remaining MRI scans for both sessions.

### 2.5 Functional connectome construction

To extract regional time‐series from the RS‐fMRI data, the UNC neonatal AAL atlas (Shi et al., 2011) consisting of 90 regions was used. First, the atlas was registered to each subject's native (T2) space using Advanced Normalization Tools (ANTs, Avants et al., 2011) with Symmetric normalization algorithm with cross‐correlation as optimization metric algorithm (SyNCC cost function). To do so, the fixed and moving images were chosen to be the T2 and the atlas intensity model images, respectively, so that the original volumes are not altered.

After obtaining the forward deformation field (i.e., going from the atlas space to the native space), the deformation was applied to the UNC tissue probability maps of Gray, White matter, and CSF (TPMs), as well as to the 90 regions atlas (label image). The output of this step is the UNC atlas and the three TPMs in the native subject's T2 space. Next, using these TPMs, the subject's T2 image is segmented using SPM8 and the subject‐specific GM, WM and CSF probability maps are obtained in the T2 space.

**FIGURE 2:**
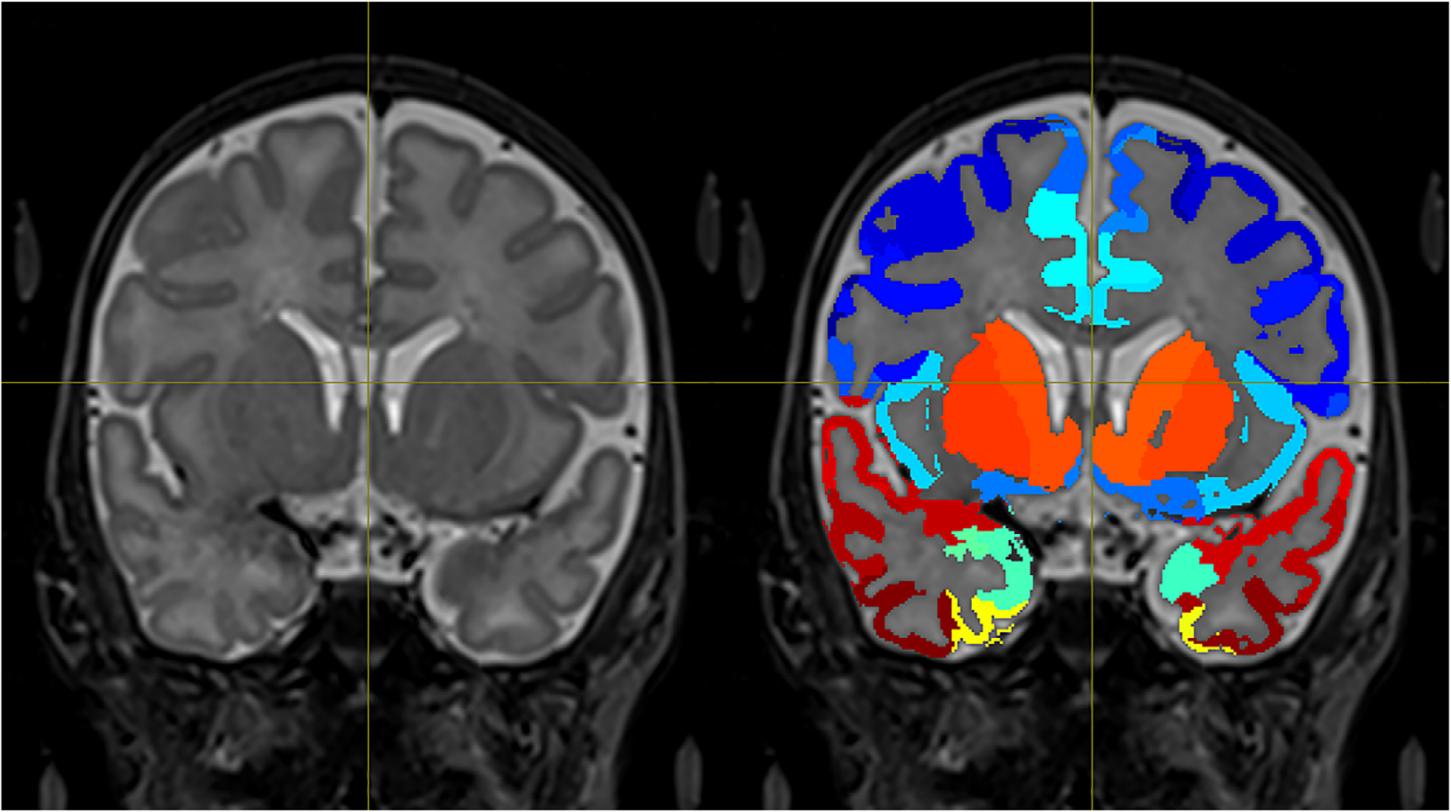
Example of the final GM‐masked atlas of one subject. Only BOLD signals from GM voxels are considered. Color coding corresponds to the atlas labeling

Furthermore, to ensure that we extract BOLD signals only from GM voxels, the UNC atlas in the native space was masked using the subject‐specific tissue probability maps obtained by the segmentation step. If the probability of one voxel being GM was less than the probability of this voxel being WM or CSF (P(GM) < P(WM) or P(GM) < P(CSF)), then this voxel was not considered as GM and it was excluded from the atlas. The output GM‐masked UNC atlas in the T2 subject space for one subject is shown in Figure 2, where the color coding corresponds to the atlas labeling.

The GM‐masked atlas was then resliced to the functional (BOLD) space and the average regional BOLD time‐courses were extracted, after performing voxel‐wise nuisance regression (CSF, WM, motion signals) and detrending, yielding a matrix with dimensions [number of volumes, 90] for each subject and each RS‐fMRI run (before and after the musical stimulus presentation). The average regional time‐courses were finally bandpass filtered ([0.01–0.1 Hz]) to discard noise components.

Lastly, the functional connectome of each subject was constructed based on the accordance measure (Meskaldji, Morgenthaler, & van de Ville, 2015) that captures coupling between brain regions. This resulted in a 90 by 90 functional connectome per run for each subject represented by a symmetric weighted matrix (see Figure 1), which can be used for connectome‐based statistical analysis and multivariate modeling.

### 2.6 Statistical connectome‐based analysis

#### 2.6.1 Investigating the short‐term effect of music listening on subsequent RS‐FC

To investigate whether the functional connectivity was altered after the presentation of a musical stimulus, connection‐wise (using the upper triangle part of the functional connectomes) one‐sided paired t‐tests (between run 2 and run 1) were performed for each group, separately. Additionally, the Cohen's *d* metric was calculated for each test to characterize the effect size and to complement the statistical analysis given that the statistical power is limited due to the small sample sizes. The statistical tests were employed to address the scientific question of interest (run 2 > run 1). A positive *t* value would indicate increased connectivity for that specific connection in run 2 (after the presentation of the musical stimuli) compared to run 1 (before the stimuli).

#### 2.6.2 Investigating the brain trace of a familiar musical stimulation in the Preterm‐Music group

To unveil changes in the functional connectivity evoked by the familiar musical stimuli (presented between the RS‐fMRI runs) in the PM group, the group‐specific *p* value vectors, resulted from the previous step (see “Investigating the short‐term effect of music listening on subsequent RS‐FC” section) were used.

First, we converted the *p* values obtained in the previous step into z‐scores ($Z_{\mathrm{PM}},Z_{\mathrm{PC}}$) encoding the effect of interest (increased functional connectivity in run 2 compared to run 1). The conversion (*p* values to z‐scores) was performed using the quantile function of the normal distribution and the z‐scores follow the standard normal distribution under the null hypothesis. Next, to explore the short‐term effects of the intervention on the functional connectivity of the Preterm‐Music group (the effect of listening to the same music), we accounted for the effect that the Preterm‐Control group experienced (i.e., listening to music for the first time during the MRI scan) by subtracting the z‐score of the PC group from the one of the PM group. We then made the resulted vector of differences normally distributed under the null:

$$\frac{Z_{\mathrm{PM}}-Z_{\mathrm{PC}}}{\sqrt{2}}∼N\left(0,1\right).$$

Here, we divided by $\sqrt{2}$ to obtain valid z‐scores with zero mean and unit standard deviation (based on the Variance Sum/Difference Law we have):

$$\text{variance}\left(Z_{\mathrm{PM}}-Z_{\mathrm{PC}}\right)=\text{variance}\left(Z_{\mathrm{PM}}\right)+\text{variance}\left(Z_{\mathrm{PC}}\right)-2\mathrm{cov}\left(Z_{\mathrm{PM}},Z_{\mathrm{PC}}\right)=2,$$

assuming that $2\mathrm{cov}\left(Z_{\mathrm{PM}},Z_{\mathrm{PC}}\right)=0$ under statistical independence of the group‐specific z‐score vectors that holds in this case (independent groups).

This enables us to define a z‐score threshold that correspond to a specific *p* value under the null (e.g., z‐threshold of 2.58 corresponds to a *p* value of .005) and to obtain significant results at the specific alpha level.

#### 2.6.3 Multivariate modeling: Exploring the dosage effect of prior music listening on the functional connectivity

To further investigate neural correlates of the music intervention effect on the brain's functional connectivity, multivariate brain outcome associations for the Preterm‐Music (PM) group, were explored using Partial Least Squares Correlation (PLSC) (Krishnan, Williams, McIntosh, & Abdi, 2011; McIntosh & Lobaugh, 2004). In this study, the outcome variable was chosen to be the actual number of times that each PM infant listened to the musical stimuli during the hospitalization that is, the occurrence of the prior musical intervention—dosage (see Table S2 for more details). The imaging variables for the PLSC analysis were brain network‐based features. For each subject in the Preterm‐Music group, a brain graph was created by treating the FC connectome as a weighted, undirected, symmetric adjacency matrix. Next, the nodal strength of all 90 vertices of the graph (brain regions) were estimated. The procedure was repeated for each run (run 1 & run 2), resulting in two vectors of nodal strengths per run for each PM infant.

Since our goal was to relate RS‐FC changes (imaging variables), induced by the musical stimulus, to the actual number of music listening episodes during hospitalization (outcome variable), the difference (delta) of the two nodal strength vectors (estimated as described above) was computed (run 2–run 1). If we would only use the brain nodal measures of run 2 (i.e., using only the post musical stimulus presentation nodal strength) then we would not ensure that we focus on pure stimuli‐induced connectivity changes. By doing so, we ensure that the outcome variable is going to be related to the actual change of brain connectivity induced by the musical stimulus. A positive delta nodal strength value indicates increased nodal strength of the corresponding brain region in run 2 compared to run 1, that is, following the musical presentation. The subject‐specific delta vectors were used as imaging variables for the PLSC analysis.

#### 2.6.4 PLSC details

The core of the PLSC method is the singular value decomposition (SVD) of the cross‐covariance matrix $R=\mathrm{US}V^{T}$ defined as $R=Y^{T}X\in R^{N_{\text{outcomes}}\times N_{\text{imaging}}}$, where **Y** is a matrix storing the outcome variables (one outcome variable in this study) for each subject as rows ($Y\in R^{N_{\text{subjects}}\times N_{\text{outcomes}}}\;$) and **X** is a matrix storing the imaging variables ($X\in R^{N_{\text{subjects}}\times N_{\text{imaging}}}$). In this study, the matrices **X** (dimension [15 × 1]) and **Y** (dimension [15 × 90]) were z‐scored across subjects prior to the construction of matrix **R**. Given that we only have one outcome variable, the estimated cross‐covariance matrix **R** has dimension [1 × 90].

The SVD of **R** results in correlation components composed of a set of outcomes (column of **U**) and brain salience weights (columns of **V**). In the present study we only have one PLSC component considering that **R** has dimension [1 × 90]. This component is associated with a singular value (value in **S**) that specifies the explained cross‐correlation along this dimension similarly to the explained variance in a Principal Components Analysis (PCA) analysis. Outcome and brain imaging salience weights (**U**, **V**) indicate how strongly each input variable contributes to the multivariate outcome‐brain correlation in this component.

To assess the statistical significance of the PLSC component, permutation testing was performed (5000 permutations) and finally, to evaluate the stability and importance of the brain and behavior saliences, bootstrapping (200 bootstrapping samples) with replacement was used as in Krishnan et al., and Zöller et al. (Krishnan et al., 2011; Zoeller et al., 2019). These two steps provide complementary information about the statistical significance of the component (permutation testing) and the stability of regional contributions (elements of the saliences) to the multivariate pattern (bootstrap resampling, McIntosh & Lobaugh, 2004), (see “PLSC Section” in Appendix S1for more details). Stable PLSC salience weights are reported in terms of bootstrapping mean and standard deviations for the statistically significant component (as assessed by the permutation testing).

Finally, we applied cross‐validation (CV) as an additional layer to strengthen the results of the PLSC analysis that reveals multivariate correlations between the intervention dose (music exposure time) and the brain imaging data for the PM group. The CV analysis aimed to further assess the stability of the latent variables (***Lx = XV**, **Ly = YU***) when tested on the left‐out data. In more detail, leave‐one‐out cross‐validation (LOOCV) was used to create folds of training and test sets. The PLSC fitting was done using the training set and then, the learnt normalization and projection on the PLSC component were applied to the left‐out test sample in order to obtain its brain and outcome scores (***Lx**, **Ly***). Then, we created two scatter plots: for each left‐out sample, we plot its brain score when using the full sample versus the one predicted when left out, and a similar plot for the outcome scores. High correlation between the cross‐validated and full model scores is indicative of the robustness of the PLSC analysis when considering left‐out samples.

## 3 RESULTS

### 3.1 Short term effect of music listening on subsequent RS‐FC

**FIGURE 3:**
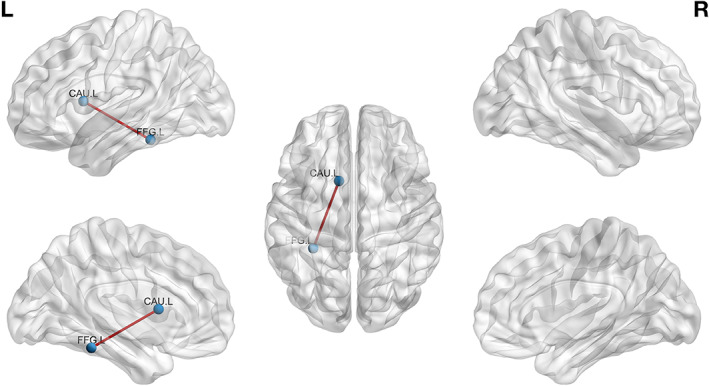
Full‐term group run 2 > run 1 differences. Edges represent connections that were increased in run 2 compared to run 1 (see Table 1)

**FIGURE 4:**
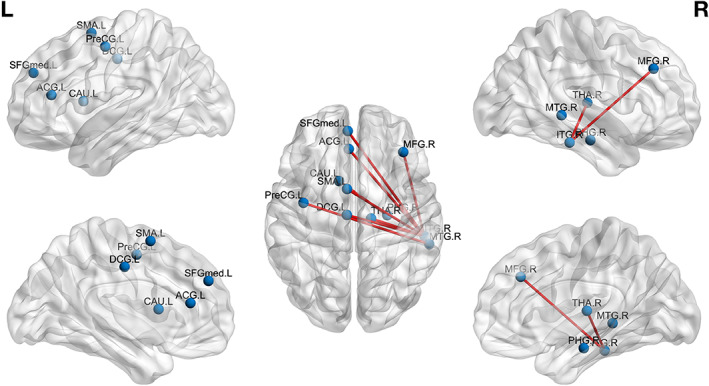
PC group: run 2 > run 1 differences. Edges represent connections that were increased in run 2 compared to run 1 (see Table 2)

**FIGURE 5:**
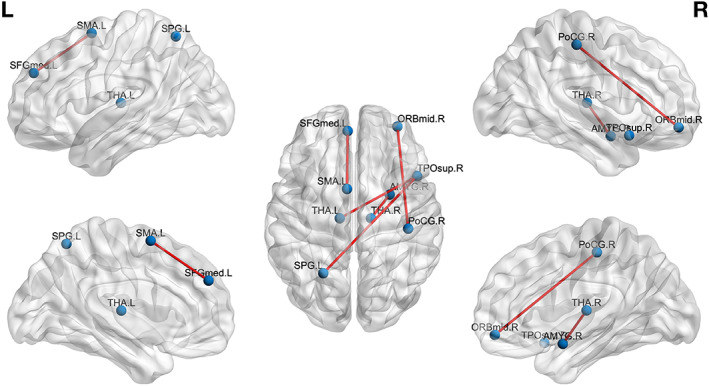
Preterm‐Music group run 2 versus run 1 differences. Edges represent connections that were increased in run 2 compared to run 1 (see Table 3)

In Tables 1, 2, 3, we report and present the connections with higher accordance in run 2 compared to run 1 for each group separately (FT, PC, PM). We report connectivity differences with corresponding *p* values <0.001 (uncorrected), due to the reduced statistical power (small sample size; *n* = 15), and we also report the Cohen's *d* metric that characterizes the effect size (magnitude of the effect) to accompany the *p* values. In Figures 3, 4, 5, we present these connections in a brain glass surface using BrainNet plotting toolbox. The connectomic‐based analysis revealed distinct effects of music listening on subsequent RS‐FC for all three groups.

**TABLE 1:** FT group: Increased functional connectivity between brain regions in run 2 compared to run 1

	Region	Region	*p* value	Cohen's *d*
1	Left fusiform gyrus	Left caudate nucleus	0.00021	0.8610

**TABLE 2:** PC group: Increased functional connectivity between brain regions in run 2 compared to run 1

	Region	Region	*p* value	Cohen's *d*
1	Right thalamus	Right inferior temporal gyrus	4.45E‐05	1.1744
2	Left superior frontal gyrus, medial	Right inferior temporal gyrus	0.00015	1.2131
3	Left median cingulate and paracingulate gyri	Right inferior temporal gyrus	0.00038	1.3858
4	Left precentral gyrus	Right middle temporal gyrus	0.00042	0.9495
5	Left supplementary motor area	Right inferior temporal gyrus	0.00049	1.5041
6	Right Parahippocampal gyrus	Left caudate nucleus	0.00079	1.0203
7	Left anterior cingulate and paracingulate gyri	Right inferior temporal gyrus	0.00079	0.9439
8	Right middle frontal gyrus	Right inferior temporal gyrus	0.00083	1.036

**TABLE 3:** PM group: Increased functional connectivity between brain regions in run 2 compared to run 1

	Region	Region	*p* value	Cohen's *d*
1	Left superior parietal gyrus	Right temporal pole: Superior temporal gyrus	0.00000966	1.217
2	Left thalamus	Right temporal pole: Superior temporal gyrus	0.00036	1.331
3	Right middle frontal gyrus, orbital part	Right postcentral gyrus	0.00071	1.513
4	Left supplementary motor area	Left superior frontal gyrus, medial	0.00074	0.833
5	Right amygdala	Right thalamus	0.00087	0.922

In the FT control group, an increased RS‐FC between the left fusiform gyrus and the left caudate nucleus was observed at rest, after music listening.

In the PC group, we observe an increase in the RS‐FC between the right inferior temporal gyrus and right thalamus, right middle frontal gyrus, medial part of the left superior frontal gyrus, left middle cingulate gyrus (MCC) and anterior cingulate gyrus (ACC), left supplementary motor area (SMA), and between right middle temporal gyrus and left precentral gyrus, as well as between right parahippocampal gyrus, and left caudate nucleus.

Finally, in the PM group, listening to music increased the RS‐FC between brain regions implicated in music, multisensory processing, familiar music processing, and processing of emotional content. In more detail, we observed an increased RS‐FC, following the music listening, between the right temporal pole and the left superior parietal gyrus and left thalamus, as well as between the right orbitofrontal region (orbital part of the middle frontal gyrus) and the right postcentral gyrus; left superior motor area and left superior medial frontal gyrus; and between the right amygdala and the right thalamus.

### 3.2 Brain trace of a familiar musical stimulus on the preterm‐music group

In this section, we report the short‐term effects of the intervention on the PM group, after accounting for the effect that the PC group experienced (see subparagraph B in “Statistical Connectome‐based Analysis” section for more details). The results were thresholded using a *z* value 2.58 that corresponds to a *p* value 0.005 for the normal distribution.

The comparison of the modulation of the RS‐FC connectivity, following the presentation of the musical stimulus, between the two preterm groups (PM group > PC group) revealed an increase RS‐FC between OFC regions and right calcarine fissure. Additionally, increased connectivity was observed between left thalamus and right temporal pole, as well as between right olfactory cortex and the right angular gyrus in favor of the PM group.

### 3.3 Dose effect of prior music listening on the functional connectivity

Based on the permutation testing, the PLSC component was found to be statistically significant (*p* = .043). The permutation null distribution is presented in Figure S2. The outcome (bottom left) and brain imaging (top) saliences (elements of U and V, respectively) of the aforementioned significant component are shown in the top part of Figure 7 (*x*‐axis: index of brain region, y‐axis: salience weight). The robust brain salience weights (highlighted with yellow in Figure 7 (top), green dots represent the bootstrapped brain salience values) can be interpreted similarly to correlation values since the data were standardized. Figure 7 (bottom right) shows, for each test sample, its brain/outcome score when using the full sample (no cross‐validation, *x*‐axis), versus the brain/outcome score predicted when left out (cross‐validated score, *y*‐axis). The high correlation between the cross‐validated and full model scores (*r* = 0.83 and *r* = 0.99 for the brain and outcome scores, respectively) further reflects the robustness of the PLSC analysis when considering left‐out samples.

Based on the PLSC results, we observe a significant *positive* correlation of the intervention occurrence (i.e., the number of music listening during hospitalization—dosage) with the positive increase of nodal strength (delta: run 2 > run 1) of L Amygdala, R putamen and L temporal pole (middle temporal gyrus). On the other hand, there is a significant *negative* correlation of the intervention frequency with the positive delta nodal strength (delta: run 2 > run 1) in R middle frontal, L Inferior frontal (triangular part), R superior frontal, L superior frontal (medial orbital), R posterior Cingulate gyrus, R hippocampus, R parahippocampal, L Inferior Occipital, R Fusiform, L Inferior parietal, R Inferior parietal, L paracentral lobule, L thalamus, and R middle temporal. These results are also presented in Figure 8 on a brain glass surface using the BrainNet plotting toolbox.

## 4 DISCUSSION

Music is known to induce emotions and associated musical memories. In adults, it is well known that complex auditory sound streams such as music, activate both working memory networks and limbic networks. We have recently shown that a familiar music is processed differently from unfamiliar music already in the newborn period. Musical memories are thought to go beyond the immediate processing of a musical stimulus. The present study aims to evaluate the effects of familiar and first‐time music listening on the subsequent RS‐FC in the brain and to assess the dose effect of prior music intervention.

### 4.1 Modulation of functional connectivity at rest after music listening

Our findings show that music listening can induce modulation of the RS‐FC in both hemispheres as early as TEA in preterm and full‐term newborns. Even if lateralization tendency has previously been observed in adults, music processing engages auditory pathways in both left and right hemispheres (Schirmer, Fox, & Grandjean, 2012). It is thus not surprising to observe increased functional connectivity between brain regions in both hemispheres as well as between the two hemispheres after music listening in our newborns. We describe the effect of music listening on subsequent RS‐FC between regions implicated in sensory functions, higher cognitive functions and emotional processing. Listening to music is a complex process for the brain, known to trigger cognitive and emotional responses with distinct neural substrates. The present findings suggest that music perception cannot be summarized only by acoustic analysis, since it also involves auditory memory, processing of interval relations and of musical syntax and semantics, as well as emotions (Koelsch, 2014).

It is also worth noting that we observed much fewer modifications by music listening of brain RS‐FC in full‐term compared to preterm newborns, which confirms prior studies in which preterm infants, due to earlier extrauterine experience, modify their brain capacity of sensory and auditory processing (Baroncelli et al., 2010). It has been shown that premature infants can process auditory stimuli and even possess the ability to process subtle changes of sounds as early as 29 weeks of gestational age (Mahmoudzadeh et al., 2013). Recent work from our research group on voice processing in preterm infants shows that preterm infants show additional cortical regions involved in voice processing in fMRI and a late mismatch response for maternal voice, considered as the first trace of a recognition process based on memory representation. This indicates an experience‐dependent maturation of brain networks in preterm infants with the early postnatal exposure to voices (Adam‐Darque et al., 2020).

Furthermore, the brain does not develop normally in the absence of external stimuli and activity of its neuronal system during the respective critical period of development (Morishita, Miwa, Heintz, & Hensch, 2010). Deprivation of sounds and specific auditory stimuli, such as vocal sounds, have been shown to prevent the auditory cortex from maturing and developing normally (Pineda et al., 2014). Additionally, preterm babies with higher endogenous spontaneous brain activity in the first 72 h of life show subsequently better brain growth at term age (Benders et al., 2015). The current results, with a significantly higher number of RS‐FC modulations after listening to music in preterm controls infants compared to full‐term infants, can be viewed as an enhanced development, based on sensory stimulation in the neonatal period, and especially sound processing.

Moreover, when comparing RS‐FC immediately before and after music listening, we observed modulation of RS‐FC in different brain regions in preterm and full‐term infants. In FT control newborns, a higher RS‐FC was observed between the left fusiform gyrus and the left caudate nucleus. The caudate nucleus is highly involved in music listening processing. Furthermore, increased activity of the caudate nucleus has been observed during rhythmic information processing (Bengtsson & Ullén, 2006) and consonant (Trost et al., 2014), pleasant (Koelsch & Skouras, 2014) and emotional arousing music processing (Mitterschiffthaler, Fu, Dalton, Andrew, & Williams, 2007; Trost, Ethofer, Zentner, & Vuilleumier, 2011). The caudate nucleus is further thought to be involved in sensorimotor behavior, such as experiencing chills during music listening (Koelsch, 2014). Therefore, the caudate nucleus is not only implicated in music processing but also in multisensory response to music listening. In the present study, full‐term newborns, after listening to music, displayed increased functional connectivity between the caudate nucleus and the fusiform gyrus, another region known to be highly implicated in visual and multisensory processing (Zhang et al., 2016). It has been shown in adult populations, that the fusiform area is activated conjointly, and increases its functional connectivity, with emotional brain regions (amygdala and insula) when music is used to enhance the emotional experience during picture or film presentation (Baumgartner, Lutz, Schmidt, & Jäncke, 2006; Pehrs et al., 2014). The fusiform gyrus thus has a crucial role in the processing of emotional multisensory stimuli. Music listening, therefore, engages multisensory processes and it is thus not surprising to observe higher functional connectivity after music listening between brain regions implicated in music and multisensory processing.

In PC infants, we observed an increase in RS‐FC between the right inferior temporal gyrus and right thalamus, right middle frontal gyrus, medial part of the left superior frontal gyrus, left middle cingulate gyrus (MCC) and anterior cingulate gyrus (ACC), left supplementary motor area (SMA), and between right middle temporal gyrus and left precentral gyrus, as well as between right parahippocampal gyrus and left caudate nucleus. As in FT, but to a greater extent, listening to music modulates postlistening RS‐FC in PC between brain regions implicated in music and multisensory stimuli processing. We observed, in both groups, a modulation of caudate nucleus RS‐FC, known, as discussed earlier, to be a multisensory processing hub network. Interestingly, we also found RS‐FC modulation in PC, but not in FT immediately after music listening between regions known to be implicated in direct music processing and linked, in adults, to previous music practice, including ACC, MCC, SMA, inferior temporal gyrus, middle frontal gyrus and superior frontal cortex. Functional connectivity in ACC and MCC has been shown to be more developed in musicians when compared to nonmusicians (Zamorano et al., 2017). Also, Hou and colleagues (Hou, Chen, & Dong, 2015) observed a higher RS‐FC between numerous brain regions, including motor areas and inferior temporal gyrus, middle frontal gyrus, and superior medial frontal cortex in adult nonmusician depending on previous music practice. As previously reported, unlike full‐term newborns, preterm infants are exposed to higher variability of ex‐utero auditory stimulations earlier, including different and unfamiliar adult voices and potentially lullabies or songs. We assumed that due to this variability in auditory extra‐uterine stimulations, they developed specific abilities to process sounds and streams of sounds, such as music, and thus activate more specific brain regions implicated in such processing during music listening.

We did not only observe modulations of RS‐FC between brain regions implicated in music processing, but also in regions linked to novel stimulus processing. In adults, watching novel visual stimuli (Blackford, Buckholtz, Avery, & Zald, 2010; Wright et al., 2003), as well as listening to an unfamiliar language (Karmonik et al., 2016), induces an increased activation in the inferior temporal cortex. In this study, we observed an increase in the RS‐FC between the inferior temporal cortex and numerous other brain regions after listening to this unknown music. We thus assume that this increased RS‐FC reflects the further processing of the unknown music that was just heard. Moreover, we observed an increased RS‐FC of the inferior temporal cortex with regions implicated both in music processing and emotional music processing. More specifically, in adults, the supplementary motor area, ACC, thalamus, middle frontal gyrus, parahippocampal gyrus, middle temporal gyrus, and caudate nucleus, are activated in response to the emotion evoked by music (Bogert et al., 2016; Bravo et al., 2019; Brown, Martinez, & Parsons, 2004; Koelsch, 2014) and to music processing (Alluri et al., 2012; Peretz & Zatorre, 2005). Also, a recent meta‐analysis showed that ACC is more activated during unfamiliar music listening (Freitas et al., 2018). We therefore conclude that in PC, novel music listening increased subsequent RS‐FC between brain regions implicated in music, novelty, and emotional processing.

### 4.2 Brain trace of a familiar musical stimulus in the preterm music group

In the PM group, listening to music modulated subsequent brain RS‐FC between brain regions implicated in music and multisensory processing, but also between brain regions known to be linked to processing of familiar music and processing of emotional content. In more detail, RS‐FC between the right temporal pole and the left superior parietal gyrus and left thalamus, as well as between the right orbitofrontal region (orbital part of the middle frontal gyrus) and the right postcentral gyrus; left superior motor area and left superior medial frontal gyrus; and between the right amygdala and the right thalamus was increased after music listening in the PM group. As mentioned previously, music processing activates motor and sensorimotor brain regions, including supplementary motor and postcentral gyrus (Gordon, Cobb, & Balasubramaniam, 2018), as well as limbic and paralimbic regions, including amygdala, orbitofrontal (OFC) regions, thalamus, and temporal pole (Koelsch, 2014). However, RS‐FC modulations between the amygdala and OFC regions were only found in PM group and not in PC or FT groups. Since the music presented between the two resting‐state runs were the same as the one used during the hospitalization of the PM infants, we infer that modulation of RS‐FC between these regions is not only due to immediate effects of music listening, but also because this music is familiar to the infants and evokes emotional memories. In adults, music familiarity increases the “liking” rate (Ali & Peynircioǧlu, 2010), and electrodermal activity (Van Den Bosch, Salimpoor, & Zatorre, 2013). Thus, music familiarity may play a role in modulating the emotional response of the listener. Furthermore, compared to unfamiliar music, familiar music activates more emotion‐related regions, including the amygdala, thalamus, supplementary motor area, temporal poles and orbitofrontal regions (Pereira et al., 2011), as well as memory‐related regions such as superior frontal and superior parietal regions (Sikka, Cuddy, Johnsrude, & Vanstone, 2015). Also, the superior frontal gyrus and thalamus have been shown, in a recent meta‐analysis, to have the highest likelihood of being activated when listening to familiar music (Freitas et al., 2018). Thus, the increased RS‐FC observed here between auditory, familiar and emotional brain regions, may reflect musical memories evoking brain activations similar to familiar music listening.

These results are strengthened by the comparison of the modulation of RS‐FC connectivity immediately after music listening between the two preterm groups (PM group > PC group). Based on our findings, we observed a greater increase in RS‐FC between OFC regions (orbital part of the right middle frontal gyrus and orbital part of the left middle and superior frontal gyrus) and right calcarine fissure, and between left thalamus and right temporal pole, as well as between right olfactory cortex and the right angular gyrus in the PM group. All these regions can be linked to the multisensory processing induced by music listening, and to familiar and emotionally arousing music processing, and therefore to associative memory and memory retrieval of the earlier experienced music (Bonnici, Richter, Yazar, & Simons, 2016). More specifically, listening to music, in the PM group, led to a greater increase of subsequent RS‐FC between the angular gyrus (a region known to participate in memory retrieval, multisensory integration and implicated in attention to relevant information (Seghier, 2013)), and brain regions implicated in visual, olfactory, cognitive and emotional processing. When comparing the PM to the PC group, PM showed a higher modulation of RS‐FC in visual brain regions (calcarine fissure and surrounding cortex) with numerous other brain regions. In animal models, early tactile interventions from birth contribute to the acceleration of the visual system development in rodents, both at behavioral and electrophysiological levels (Cancedda et al., 2004; Landi et al., 2007). Another preliminary study on early massage intervention in a preterm population showed a difference in the maturation of visual function in infants who received massages in comparison to those who received standard care (Guzzetta et al., 2009). Music exposure during early ex‐utero life may thus have left multisensory brain traces that are reactivated by familiar music listening through musical memory retrieval. These results are also in line with the increased RS‐FC of the angular gyrus in adults after listening to pleasant, soothing, and familiar music (Garza‐Villarreal et al., 2015). Furthermore, we also found a greater increase of RS‐FC between brain regions known to be linked to familiarity and pleasantness (including thalamus, orbitofrontal, and superior frontal regions), which is consistent with the fact that these infants already knew the music (Freitas et al., 2018; Pereira et al., 2011). Previously, using a Psychophysiological Interaction Analysis, we observed an increased functional connectivity in preterm infants' brain between regions implicated in music processing, familiarity and pleasantness during known music listening (Lordier et al., 2018, 2018). Here, we further show that increased functional connectivity linked to this familiar and pleasant music listening is present at rest, minutes after music listening, indicating the presence of an associative memory process for the familiar music (Platel et al., 2003).

Preterm infants with early music intervention listened to music that was especially composed for them during their hospitalization. During weeks, this music was used to punctuate the day of these preterm infants. It is now well known that events, and even more repeated and meaningful events, leave a trace on brain structure and function. Memory encoding and retrieval processes, including musical memories, lead to the joint activation of different populations of neurons. This eventually results in an increase in the synaptic strength between this ensemble of neurons, which then finally leads to the formation of an engram cell ensemble, an old theory of memory formation and retrieval that still has value today in neuroscience (Josselyn & Tonegawa, 2020). Any percept, or emotional memory formation, is represented by a specific group of engram cells (or memory associative cells) i.e., a special cell ensemble. Emotional associate memories have been shown to be processed through such ensembles or engrams of specific groups of neurons (Bocchio, Nabavi, & Capogna, 2017). Retrieval of memories involves cues, external sensory (e.g., in our experiment, musical tones) or internally generated stimuli (e.g., in our experiment, associate emotions to music listening), and reawakening specific neuronal engrams (Frankland, Josselyn, & Köhler, 2019). The associated engram cells or memory associative cells in this special group are distributed over broad cortical and subcortical areas rather than in a special brain region, (Josselyn, Köhler, & Frankland, 2015) making it possible to capture such functional brain states using in vivo approaches of functional MRI. Supporting this, Richiardi and colleagues (Richiardi et al., 2015) showed a correlation of functional resting‐state dynamics and synaptic gene expression, while Canals and colleagues (Canals, Beyerlein, Merkle, & Logothetis, 2009) showed recruitment of neocortical and limbic circuits after hippocampal changes in synaptic strengths. Activity in engram cells is persistent and it is still observed during subsequent sleep or wakeful rest. Resting‐state fMRI may be used to observe the reoccurrence of neural activity patterns between engram cells, produced by previous experience. For instance, Hamano and colleagues recently studied the motor engram as a dynamic change of the cortical network during learning as assessed by fMRI (Hamano et al., 2020).

**FIGURE 6:**
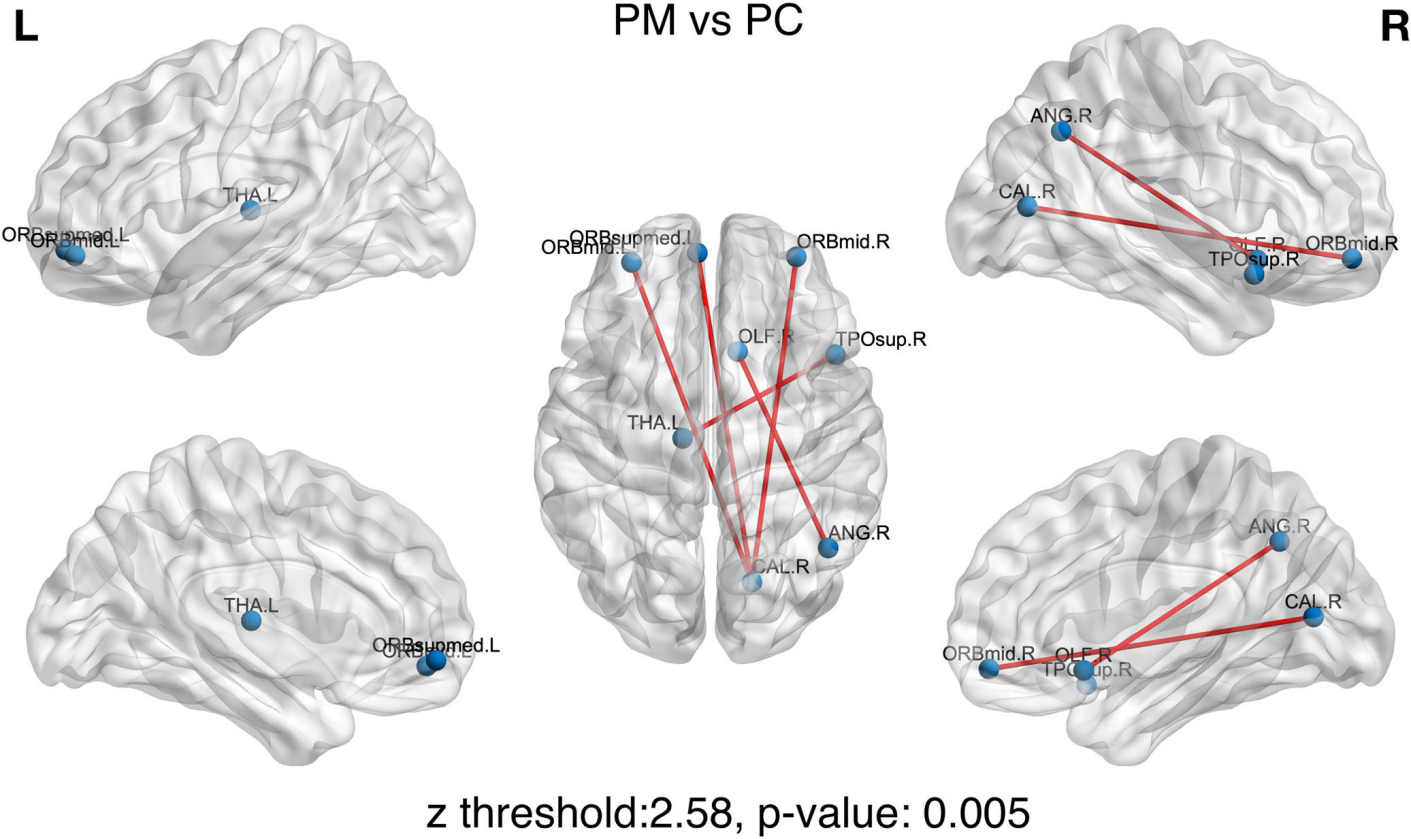
The short‐term effects of intervention on the PM group after accounting for the effect that the PC group experienced. Increased functional connectivity between brain regions in run 2 compared to run 1

Thus, experience leads to traces in neuronal plasticity, an engram (Josselyn et al., 2015), as well as in psychological traces (Ansermet, 2019; Escobar, Ansermet, & Magistretti, 2017). Early music intervention, during the first weeks of life may leave both psychic and neuronal traces in PM infants. However, if the modulation of subsequent RS‐FC observed here is the reflection of traces left by music exposure (training) during hospitalization, intervention dose effects should be observed (Figure 6 and Table 4).

**TABLE 4:** The short‐term effects of intervention on the PM group after accounting for the effect that PC experienced: Increased functional connectivity between brain regions in run 2 compared to run 1

Region	Region	*z*‐score difference
Right middle frontal gyrus, orbital part	Right Calcarine fissure and surrounding cortex	3.341
Left thalamus	Right temporal pole: Superior temporal gyrus	2.918
Left middle frontal gyrus, orbital part	Right Calcarine fissure and surrounding cortex	2.803
Right olfactory cortex	Right angular gyrus	2.672
Left superior frontal gyrus, medial orbital	Right Calcarine fissure and surrounding cortex	2.665

**FIGURE 7:**
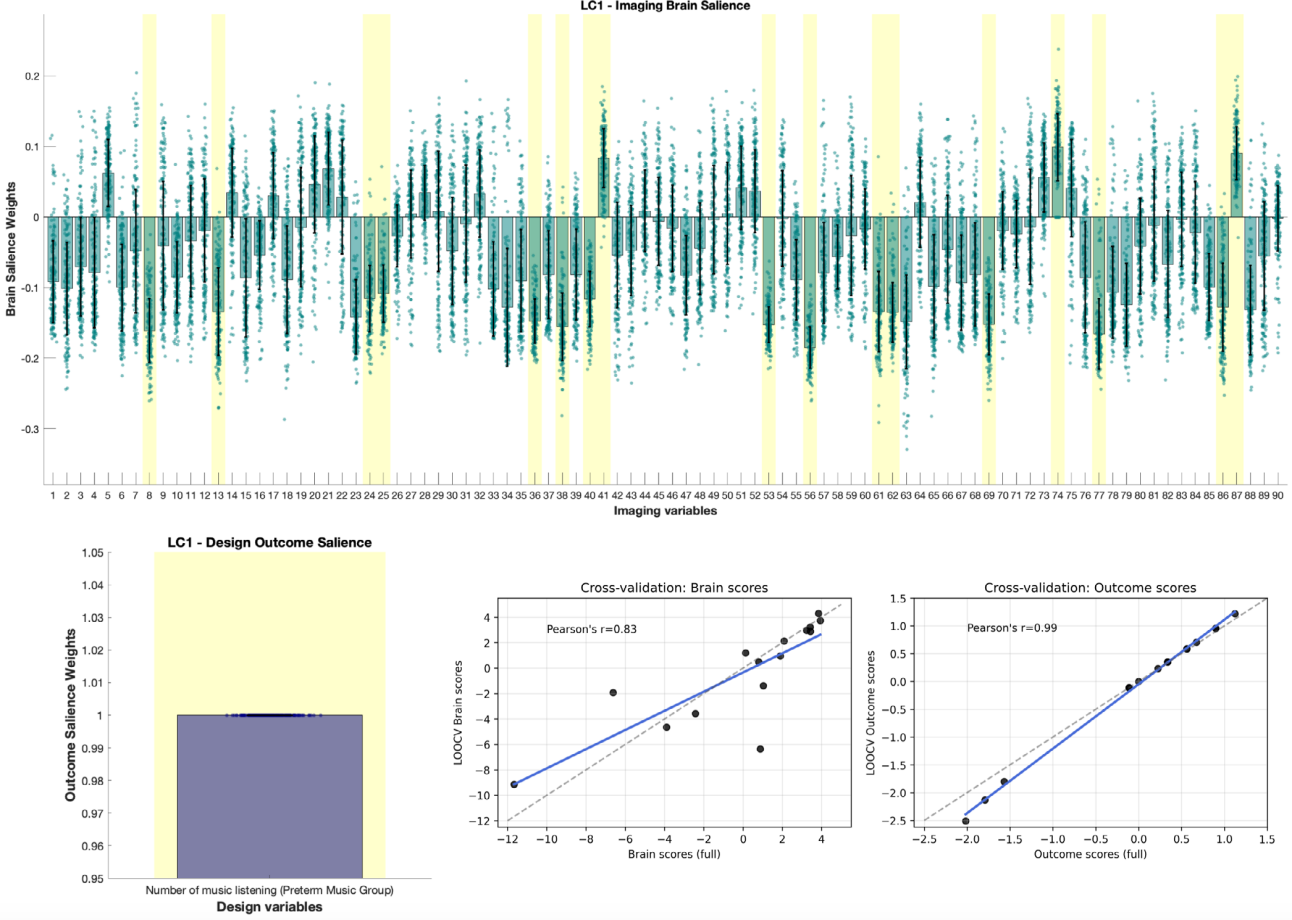
Partial least squares correlation (PLSC) results for the effect of the intervention in PM group (number of listening to the musical stimulus during the hospitalization). The outcome (weight in U; bottom left) and brain imaging saliences (weights in V; top) of the significant PLSC component (*p* = .043, 5000 permutations) are presented. Error bars indicate bootstrapping 5th to 95th percentiles and robust results are indicated by a yellow background. The exact values of bootstrap mean percentiles are reported in Table S4. Bar heights correspond to the salience weights of the significant PLSC component. Each bar corresponds to one brain region (*n* = 90 in the UNC atlas) and depicts the brain regional contributions (saliences weights) to the multivariate pattern/correlation. Bottom right scatter plots: for each left‐out sample, we plot its brain and outcome score when using the full sample (x‐axis), versus the one predicted when left out (leave‐one‐out cross‐validation, y‐axis). The points represent the PM subjects (*n* = 15)

**FIGURE 8:**
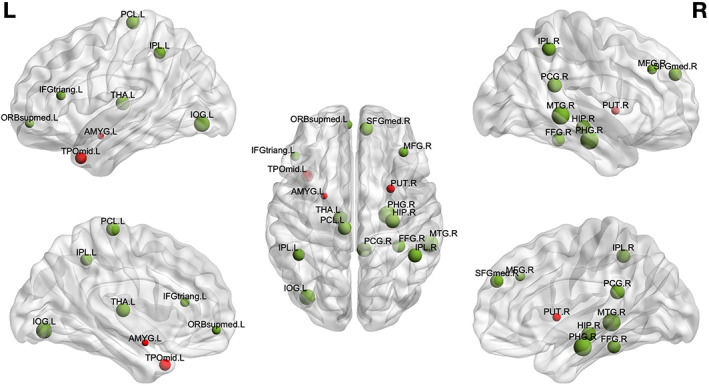
Brain glass‐surface representation of the Partial least squares correlation (PLSC) results for the effect of the intervention in PM group (number of listening to the musical stimulus during the hospitalization). Red color: nodes with significant positive brain‐outcome correlation. Green color: nodes with significant negative brain‐outcome correlation. The size of the nodes is proportional to the delta nodal strength (run 2 > run 1)

### 4.3 Dose effect of prior music listening on the functional connectivity

To further study the brain traces of the previous music exposure, we used Partial Least Squares Correlation (PLSC), a well‐known method for the investigation of multivariate relationships between outcome measures and brain imaging data (Krishnan et al., 2011; McIntosh & Lobaugh, 2004).

Based on the revealed multivariate relationships, we observed a significant positive correlation of the occurrence of the prior music intervention during hospitalization (i.e., the frequency of the intervention—the dosage), with the delta nodal strength of run 2 versus run1 of left amygdala, right putamen and left temporal gyrus. This means that following the presentation of the musical stimuli, the higher the intervention occurrence (during the NICU stay), the higher the delta change in RS‐FC (delta nodal strength: run 2–run 1) in these brain regions. These results are in line with previously reported results where we showed that music listening during NICU hospitalization improves music processing in preterm newborns (Lordier et al., 2018, 2018), observing an increased functional connectivity in preterm infants that underwent an early musical intervention between the auditory cortex and brain regions involved in music features processing (caudate, putamen, superior temporal gyrus), as well as in musical expertise (superior temporal gyrus and middle cingulate cortex). Furthermore, the amygdala and temporal cortices have been characterized as the brain correlates of music‐evoked emotions (Koelsch, 2014). Thus, the present results suggest that in PM infants, a higher dose of prior music listening correlates positively with an increase in RS‐FC between brain regions implicated in multisensory and emotion processing (following the musical stimulus). Along this line, an increased intervention frequency during hospitalization (dose of musical training) may have reinforced the effects of music listening both in terms of expertise in music features processing and in terms of forming associative memories including emotions. During hospitalization, the music intervention was put immediately before or after interaction with their parents or nurses, accompanying meaningful emotional events. Thus, the music intervention left neuronal traces in the preterm infants' brain, and listening again to this familiar music reactivated engram cells, leading to a modulation of functional connectivity we observed during subsequent rest.

On the other hand, we also observed a negative correlation between the occurrence of the prior musical intervention (dose) and the delta nodal strength (run 2–run 1) in right medial frontal, left inferior frontal (triangular part), right superior frontal, left superior frontal (medial orbital), right posterior cingulate gyrus, right hippocampus, right parahippocampal, left inferior occipital, right fusiform, left inferior parietal, right inferior parietal, left paracentral lobule, left thalamus, and right middle temporal. This indicates that the higher the musical intervention occurrence, the lower the delta RS‐FC change (delta nodal strength: run 2–run 1) in these regions, following the presentation of the musical stimuli. Interestingly, most of these regions, such as the posterior cingulate cortex, inferior parietal, prefrontal, middle/superior frontal, hippocampal, and temporal areas are part of the well‐known “Default Mode Network” (DMN) (Koshino, Minamoto, Yaoi, Osaka, & Osaka, 2014). In adults, it has been reported that the DMN shows low intrasubject variability (Mueller et al., 2013) and tends to remain stable in terms of connectivity changes. Moreover, the DMN is stable and mainly engaged under internal thought processes, mind wandering (Mason et al., 2007), and even during receptive music listening (Kay, Meng, DiFrancesco, Holland, & Szaflarski, 2012). DMN is thought to have an important role in the performance of these tasks because it deactivates during cognitively demanding tasks (Raichle et al., 2001). At rest, the posterior cingulate cortex shows functional connectivity with both central executive network (CEN) and DMN. But during a cognitively demanding task, ventral and dorsal posterior cingulate functional connectivity are modulated in opposite directions with the dorsal part becoming more integrated with the DMN and more anticorrelated with CEN (Leech, Kamourieh, Beckmann, & Sharp, 2011). The posterior cingulate cortex is hence thought to be implicated in the switching between CEN and DMN. Along these lines, we assume that this downregulation of the regions of the DMN, as the occurrence of the prior musical intervention (dosage) increases, is linked to the fact that music exposure has led to neuronal and psychic traces, and repeatedly listening to this music may have reactivated cognitive brain processes associated with the retrieval of familiarity, memory, and emotions. In other words, the more the PM infants listened to the music during the NICU stay, the more they engage cognitive brain processes and disengage regions of the DMN.

## 5 CONCLUSIONS

To the best of our knowledge, this study is the first to assess both the effect of music listening on the subsequent RS‐FC in preterm and full‐term newborns and musical memories through RS‐FC modulation. First, we showed that all infants experienced changes in their RS‐FC after music listening, mostly between brain regions implicated in music perception, including multisensory processing and emotion. Also, we showed that preterm infants who were previously repeatedly exposed to music increased their RS‐FC between brain regions known to be involved in associative memory and multisensory processing. We assume that modulation of subsequent RS‐FC reflects reactivation of memory associative cells, an ensemble of neurons with increased synaptic connections due to previous exposure to a repeated, pleasant and salient piece of music.

The study further indicates a brain correlate of musical memory engrams in preterm infants that listened to music during the hospital stay with a dose‐dependent effect on this modulation. These last findings confirm and extend our previous results on the preterm infant's ability to process, recognize, and activate brain regions associated with memory and familiar music processing.

## 6 LIMITATIONS

The major limitation of this study is the small sample size. As a consequence, the statistical power is limited and the connection‐wise comparisons were not corrected for multiplicity. However, a strict uncorrected *p* value threshold was applied in all cases (i.e., *p* = .001). To accompany the *p* values, we also estimated the Cohen's *d* to quantify the effect size and complement our findings. *p* values are indicative of the presence of an effect, whereas Cohen's *d* informs us about the presence of thus effect. All effect sizes were above 0.8, meaning that there is a large impact on all reported cases. Furthermore, multiplicity correction on a small sample size could potentially lead to the absence of some important underlying clinically relevant effects due to the lack of statistical power. We recognize that this is a weakness of the study, however, this is an exploratory analysis and not a confirmatory analysis. Finally, the reported PLCS results were statistically significant (*p* < .05 based on permutation testing).

## AUTHOR CONTRIBUTIONS

Serafeim Loukas: Formal analysis, Methodology, Visualization, Methods Conceptualization, Writing‐Original Draft, Writing‐Review & Editing. Lara Lordier: Data Acquisition, Writing‐Original Draft, Writing‐Review & Editing. Djalel‐E. Meskaldji: Methodology, Writing‐Review, Supervision, Methods Conceptualization. Manuela Filippa: Writing‐Review & Editing. Joana Sa de Almeida: Methods Conceptualization, Editing. Dimitri Van De Ville: Writing‐Review & Editing, Supervision. Petra S. Hüppi: Conceptualization, Writing‐Review & Editing, Supervision, Project administration, Resources, Funding acquisition.

## Supporting information


**APPENDIX S1**: Supporting Information

## Data Availability

All data were acquired in the context of the research project approved by the ethical committee. The patient consent form did not include any clause for re‐using or sharing the data. It stated explicitly that all data (clinical and imaging) would not be used for any other aim apart from the present research study and would not be shared with third parties.
