# Supplementary material for: Musical memories in newborns: A resting‐state functional connectivity study
Source: Hum Brain Mapp. 2021 Nov 5;43(2):647–64. doi: 10.1002/hbm.25677 (PMC8720188; doi:10.1002/hbm.25677)
Supplement: Supplementary file 1 — APPENDIX S1: Supporting Information [file HBM-43-647-s001.docx]

# Supplementary material

# Musical memories in newborns: A resting-state functional connectivity study

Serafeim Loukas^1, 2,4 *, ¥^, Lara Lordier^1, ¥^, Djalel-E. Meskaldji^1, 3^, Manuela Filippa^1^, Joana Sa de Almeida^1^, Dimitri Van De Ville ^2,4^ and Petra S. Hüppi ^1^

^1^ Division of Development and Growth, Department of Pediatrics, University of Geneva, Geneva, Switzerland

^2^ Institute of Bioengineering, Ecole Polytechnique Fédérale de Lausanne (EPFL), Lausanne, Switzerland

^3^ Institute of Mathematics, Ecole Polytechnique Fédérale de Lausanne (EPFL), Lausanne, Switzerland

^4^ Department of Radiology and Medical Informatics, University of Geneva, Geneva, Switzerland

* Corresponding author: [Petra.Huppi@hcuge.ch](mailto:Petra.Huppi@hcuge.ch)

^¥^ These authors contributed equally.

**Figure S1**: The enrollment procedure and further information of the exclusion criteria.

**Table S1**: Population Characteristics

|  | PM | PC | FT |
| --- | --- | --- | --- |
| N | **15** | **15** | **16** |
| GA birth (weeks) | 29.16 ± 2.14 | 28.95 ± 1.83 | 39.50 ± 1.08 |
| GA at scan (weeks) | 40.21 ± 0.55 | 40.50 ± 0.77 | 39.78 ± 1.05 |
| Socioeconomic status (SES) | 6.46 ± 3.54 | 6.34 ± 3.47 | 4.53 ± 3.06 |
| Birth weight (g) | 1203.67 ± 351 | 1161 ± 287 | 3333.43 ± 334 |
| Cranial perimeter at birth (cm) | 27.05 ± 2.82 | 26.17 ± 2.25 | 34.46 ± 1.14 |
| Height at birth (cm) | 37.84 ± 3.20 | 36.47 ± 4.02 | 49.68 ± 1.49 |

**Table S2**: Intervention details for the Preterm-Music group.

| PM (n=15) | |
| --- | --- |
| # of music listening during hospitalization | |
| Subject_001 | 30 |
| Subject_002 | 31 |
| Subject_006 | 7 |
| Subject_007 | 28 |
| Subject_008 | 33 |
| Subject_012 | 25 |
| Subject_016 | 33 |
| Subject_028 | 11 |
| Subject_032 | 30 |
| Subject_044 | 9 |
| Subject_047 | 24 |
| Subject_048 | 24 |
| Subject_051 | 28 |
| Subject_052 | 35 |
| Subject_054 | 27 |
| Mean (std) | **25 (8.92)** |

**Table S3**: Regions of interest included in AAL-atlas.

| Table S3. Regions of interest included in AAL atlas | | |
| --- | --- | --- |
| Labels | Abbreviation used in figures | Regions full name |
| 1 | PreCG | Precental gyrus |
| 2 | SFGdor | Superior frontal gyrus, dorsolateral |
| 3 | ORBsup | Superior frontal gyrus, orbital part |
| 4 | MFG | Middle frontal gyrus |
| 5 | ORBmid | Middle frontal gyrus, orbital part |
| 6 | IFGoperc | Inferior frontal gyrus, opercular part |
| 7 | IFGtriang | Inferior frontal gyrus, triangular part |
| 8 | ORBinf | Inferior frontal gyrus, orbital part |
| 9 | ROL | Rolandic operculum |
| 10 | SMA | Supplementary motor area |
| 11 | OLF | Olfactory cortex |
| 12 | SFGmed | Superior frontal gyrus, medial |
| 13 | ORBsupmed | Superior frontal gyrus, medial orbital |
| 14 | REC | Gyrus rectus |
| 15 | INS | Insula |
| 16 | ACG | Anterior cingulate and paracingulate gyri |
| 17 | DCG | Median cingulate and paracingulate gyri |
| 18 | PCG | Posterior cingulate gyrus |
| 19 | HIP | Hippocampus |
| 20 | PHG | Parahippocampal gyrus |
| 21 | AMYG | Amygdala |
| 22 | CAL | Calcarine fissure and surrounding cortex |
| 23 | CUN | Cuneus |
| 24 | LING | Lingual gyrus |
| 25 | SOG | Superior occipital gyrus |
| 26 | MOG | Middle occipital gyrus |
| 27 | IOG | Inferior occipital gyrus |
| 28 | FFG | Fusiform gyrus |
| 29 | PoCG | Postcentral gyrus |
| 30 | SPG | Superior parietal gyrus |
| 31 | IPL | Inferior parietal, but supramarginal and angular gyri |
| 32 | SMG | Supramarginal gyrus |
| 33 | ANG | Angular gyrus |
| 34 | PCUN | Precuneus |
| 35 | PCL | Paracentral lobule |
| 36 | CAU | Caudate nucleus |
| 37 | PUT | Lenticular nucleus, putamen |
| 38 | PAL | Lenticular nucleus, pallidum |
| 39 | THA | Thalamus |
| 40 | HES | Heschl gyrus |
| 41 | STG | Superior temporal gyrus |
| 42 | TPOsup | Temporal pole: superior temporal gyrus |
| 43 | MTG | Middle temporal gyrus |
| 44 | TPOmid | Temporal pole: middle temporal gyrus |
| 45 | ITG | Inferior temporal gyrus |

### **PLSC implementation details**

The core of the PLSC method is the singular value decomposition (SVD) of the cross-covariance matrix $\boldsymbol{R}\mathbf{=}\boldsymbol{US}\boldsymbol{V}^{\boldsymbol{T}}$ defined as $\boldsymbol{R}\mathbf{=}\boldsymbol{Y}^{\boldsymbol{T}}\boldsymbol{X}\boldsymbol{\in}\mathbb{R}^{\boldsymbol{N}_{\boldsymbol{outcomes}} \boldsymbol{x} \boldsymbol{N}_{\boldsymbol{imaging}}}$ , where **Y** is a matrix storing the outcome variables (one outcome variable in this study) for each subject as rows ($\boldsymbol{Y}\boldsymbol{\in}\mathbb{R}^{\boldsymbol{N}_{\boldsymbol{subjects}} \boldsymbol{x} \boldsymbol{N}_{\boldsymbol{outcomes}}}$) and **X** is a matrix storing the imaging variables ($\boldsymbol{X}\boldsymbol{\in}\mathbb{R}^{\boldsymbol{N}_{\boldsymbol{subjects}} \boldsymbol{x} \boldsymbol{N}_{\boldsymbol{imaging}}}$). In this study, the cross-covariance matrix **R** is built using the z-scored (across subjects) matrices **X** and **Y** (**μ**=0, **σ**=1). Given that we only have one outcome variable in this study, the cross-covariance matrix **R** has dimension [1 x 90].

The SVD of **R** results in correlation components each composed of a set of outcomes (columns of **U**) and brain salience weights (columns of **V**). In the present study we only have one PLSC component since **R** has dimension [1 x 90] as explained above. Additionally, this component is associated with a corresponding singular value (stored on the diagonal of **S**) that specifies the explained correlation along this dimension. Outcome (**U**) and brain imaging (**V**) salience weights indicate how strongly each input variable contributes to the multivariate outcome brain correlation in this certain component. In this study the weights range lies between −1 and 1 since we normalized the data (z-scoring of outcome and brain network input features across subjects) before the computation of the cross-covariance matrix **R** and thus, these salience weights can be interpreted similarly to correlation values.

To assess the statistical significance of this component, permutation testing was performed. Briefly, the rows of $\boldsymbol{Y}\in\mathbb{R}^{N_{subjects} x N_{outcome}}$ (i.e., the outcome variable’s elements) were permuted 5000 times and within each iteration, the permuted **R_perm_, U_perm_, S_perm_, V_perm_** were estimated. Next, the p-value was estimated from the permutation null distribution by counting the number of permuted singular values (**S_perm_**) above the observed singular value (***S***) of the PLSC component, in line with common practice and guidelines for PLSC (Krishnan et al., 2011).

To evaluate the stability of brain and outcome salience weights (i.e., the elements of the matrices **U** and **V**) , 200 bootstrap samples of **X** and **Y** with replacement were performed (sampling, following the guidelines from Krishnan et al., 2011) and the PLSC analysis was repeated obtaining the bootstrapped outcome and brain salience weights.

Finally, we applied leave-one-out cross-validation to further assess the stability of the latent variables when tested on the left-out data. Within each fold, a training set and a testing set (single left-out sample) were created. The PLSC fitting was done using the training set and then, the training normalization and projection on the PLSC component were applied to the test sample (left-out sample) to obtain its brain and outcome scores.

**Permutation testing for the PLSC analysis**

To assess the statistical significance of the computed PLSC component, 5000 permutation tests were performed. The p-value was estimated from the permutation null distribution by counting the number of times that the *permuted* singular values (*S_p_*) were above the *observed true* singular value (*S*) of the PLSC component, in line with the common guidelines for PLSC (Krishnan et al., 2011). The permutation null distribution is shown in below where on y-axis we have the frequency and on x-axis the singular values obtained by the permutation testing. The red vertical line represents the true observed singular value of the PLSC component of the empirical data, which was found to be statistically significant based on the permutation testing (p=0.043).


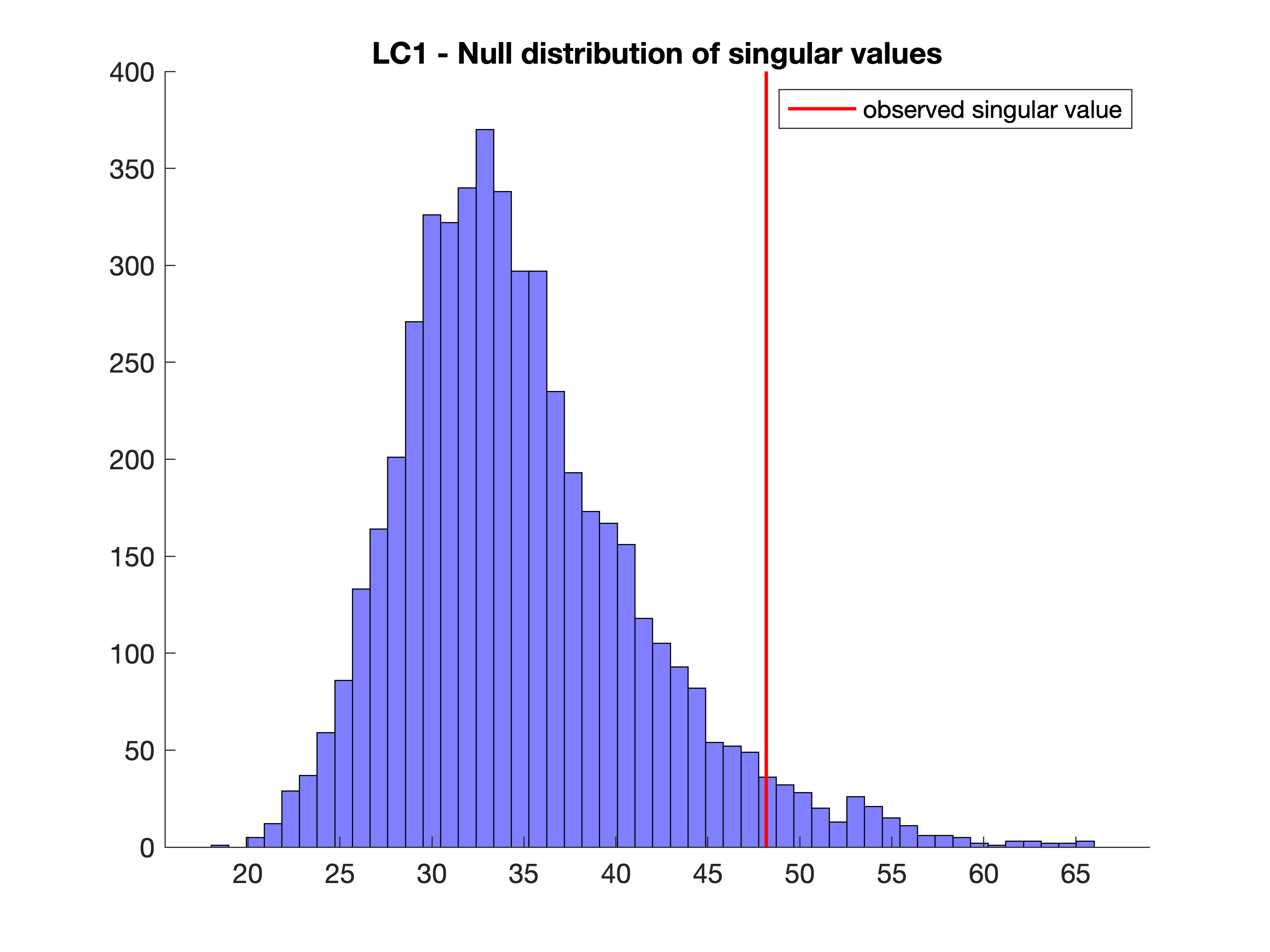


**Figure S2: The permutation null distribution**. The histogram of the null distribution of the singular values is presented among with the observed (red line) singular value of the significant latent component. Y-axis represents frequency and x-axis the singular values obtained by the permutation testing.

**Table S4**: Exact values of mean bootstrap weights and 5th to 95th percentiles for the identified statistically significant PLSC component (LC1).

| Mean bootstrapped brain salience weights | Lower bound of CI | Upper bound of CI |
| --- | --- | --- |
| -0.092 | -0.167 | 0.064 |
| -0.101 | -0.219 | 0.03 |
| -0.07 | -0.17 | 0.092 |
| -0.079 | -0.172 | 0.121 |
| 0.062 | -0.05 | 0.138 |
| -0.1 | -0.2 | 0.017 |
| -0.048 | -0.176 | 0.157 |
| -0.161 | -0.248 | -0.073 |
| -0.041 | -0.173 | 0.133 |
| -0.085 | -0.175 | 0.011 |
| -0.034 | -0.171 | 0.097 |
| -0.019 | -0.154 | 0.108 |
| -0.134 | -0.233 | -0.011 |
| 0.034 | -0.12 | 0.127 |
| -0.086 | -0.207 | 0.069 |
| -0.054 | -0.169 | 0.021 |
| 0.03 | -0.103 | 0.136 |
| -0.089 | -0.218 | 0.067 |
| -0.015 | -0.154 | 0.133 |
| 0.046 | -0.127 | 0.142 |
| 0.069 | -0.047 | 0.144 |
| 0.028 | -0.152 | 0.137 |
| -0.141 | -0.202 | 0.02 |
| -0.116 | -0.2 | -0.006 |
| -0.109 | -0.177 | -0.014 |
| -0.027 | -0.134 | 0.039 |
| 0.004 | -0.143 | 0.098 |
| 0.034 | -0.058 | 0.102 |
| 0.008 | -0.171 | 0.14 |
| -0.048 | -0.167 | 0.108 |
| -0.009 | -0.174 | 0.126 |
| 0.033 | -0.091 | 0.138 |
| -0.102 | -0.168 | 0.098 |
| -0.128 | -0.204 | 0.142 |
| -0.09 | -0.186 | 0.073 |
| -0.148 | -0.199 | -0.077 |
| -0.082 | -0.166 | 0.046 |
| -0.156 | -0.229 | -0.05 |
| -0.081 | -0.183 | 0.04 |
| -0.116 | -0.195 | -0.041 |
| 0.083 | 0.005 | 0.159 |
| -0.054 | -0.212 | 0.08 |
| -0.047 | -0.193 | 0.065 |
| 0.009 | -0.126 | 0.116 |
| -0.006 | -0.114 | 0.121 |
| -0.016 | -0.135 | 0.1 |
| -0.083 | -0.19 | 0.041 |
| -0.045 | -0.149 | 0.086 |
| -0.004 | -0.127 | 0.144 |
| 0.005 | -0.125 | 0.128 |
| 0.041 | -0.091 | 0.127 |
| 0.037 | -0.078 | 0.14 |
| -0.153 | -0.197 | -0.096 |
| -0.002 | -0.134 | 0.122 |
| -0.089 | -0.187 | 0.018 |
| -0.185 | -0.236 | -0.123 |
| -0.079 | -0.169 | 0.116 |
| -0.056 | -0.129 | 0.044 |
| -0.026 | -0.163 | 0.141 |
| -0.017 | -0.144 | 0.077 |
| -0.134 | -0.218 | -0.006 |
| -0.135 | -0.192 | -0.026 |
| -0.148 | -0.29 | 0.01 |
| 0.021 | -0.103 | 0.139 |
| -0.098 | -0.216 | 0.057 |
| -0.046 | -0.155 | 0.112 |
| -0.093 | -0.184 | 0.059 |
| -0.081 | -0.169 | 0.11 |
| -0.152 | -0.23 | -0.065 |
| -0.019 | -0.15 | 0.079 |
| -0.024 | -0.139 | 0.05 |
| -0.014 | -0.144 | 0.139 |
| 0.056 | -0.038 | 0.133 |
| 0.099 | 0 | 0.181 |
| 0.041 | -0.129 | 0.126 |
| -0.086 | -0.205 | 0.063 |
| -0.165 | -0.22 | -0.01 |
| -0.107 | -0.185 | 0.101 |
| -0.124 | -0.207 | 0.03 |
| -0.041 | -0.173 | 0.082 |
| -0.012 | -0.134 | 0.143 |
| -0.067 | -0.188 | 0.083 |
| -0.003 | -0.154 | 0.106 |
| -0.022 | -0.129 | 0.122 |
| -0.099 | -0.166 | 0.003 |
| -0.128 | -0.229 | -0.003 |
| 0.09 | 0.028 | 0.17 |
| -0.132 | -0.219 | 0.05 |
| -0.055 | -0.195 | 0.095 |
| -0.002 | -0.137 | 0.066 |

### Statistical tests for clinical variables

**Table S5**: Statistical tests for clinical variables.

| Clinical Variables | Hypothesis Testing | Conclusion | Critical value & p-value |
| --- | --- | --- | --- |
| Gender | FT vs PM vs PC | No significant difference | X2(2) = 0.035, p-value = 0.982 |
| Number of times with headphones | PM versus PC | No significant difference | F(1,29) = 0.147, p-value = 0.703 |
| Number of images (run 1) | FT vs PM vs PC | No significant difference | F(2,43) = 1.806, p-value = 0.176 |
| Number of images (run 2) | FT vs PM vs PC | No significant difference | F(2,43) = 1.259, p-value = 0.294 |
| Coil type | FT vs PM vs PC | No significant difference | F(2,43) = 0.370, p-value = 0.692 |
| GA birth | PM versus PC | No significant difference | F(1,29) = 0.082, p-value = 0.776 |
| GA MRI | FT vs PM vs PC | No significant difference | F(2,43) = 2.687, p-value = 0.079 |
| Socioeconomic Status | PM vs PC | No significant difference | F(1,29) = 0.0108, p-value = 0.917 |
| Birth Weight (g) | PM vs PC | No significant difference | F(1,29) = 0.132, p-value = 0.718 |
| Cranial Perimeter Birth (cm) | PM vs PC | No significant difference | F(1,29) = 0.594, p-value = 0.447 |
| Height birth (cm) | PM vs PC | No significant difference | F(1,29) = 1.05, p-value = 0.312 |
| Intrauterine growth restriction (yes/no) | PM vs PC | No significant difference | X2(1) = 0.0, p-value = 1.0 |
| Microcephaly (yes/no) | PM vs PC | No significant difference | X2(2) = 3.012, p-value = 0.221 |
| Chorioamnionitis (yes/no) | PM vs PC | No significant difference | X2(1) = 0.370, p-value = 0.542 |
| Asphyxia (yes/no) | PM vs PC | No significant difference | X2(1) = 3.334, p-value = 0.067 |
| BPD (yes/no) | PM vs PC | No significant difference | X2(1) = 0.681, p-value = 0.408 |
| IVH (yes/no) | PM vs PC | No significant difference | X2(1) = 0.240, p-value = 0.624 |
| Sepsis (yes/no) | PM vs PC | No significant difference | X2(2) = 1.366, p-value = 0.505 |
| NEC (yes/no) | PM vs PC | No significant difference | X2(1) = 1.034, p-value = 0.309 |
| Patent ductus arteriosus (yes/no) | PM vs PC | No significant difference | X2(1) = 0.370, p-value = 0.542 |

* ANOVA for numerical variables

* Chi-squared test for categorical variables
